# Real-Time High-Resolution MRI Endoscopy at up to 10 Frames per Second

**DOI:** 10.34133/2021/6185616

**Published:** 2021-02-17

**Authors:** Xiaoyang Liu, Parag Karmarkar, Dirk Voit, Jens Frahm, Clifford R. Weiss, Dara L. Kraitchman, Paul A. Bottomley

**Affiliations:** ^1^Department of Electrical and Computer Engineering, Johns Hopkins University, USA; ^2^The Division of MR Research, Department of Radiology, Johns Hopkins University, Baltimore, Maryland, USA; ^3^Biomedizinishe NMR, Max-Plank-Institut fur Biophysikalische Chemie, Gottingen, Germany; ^4^The Division of Interventional Radiology, Department of Radiology, Johns Hopkins University, Baltimore, Maryland, USA

## Abstract

*Objective*. Atherosclerosis is a leading cause of mortality and morbidity. Optical endoscopy, ultrasound, and X-ray offer minimally invasive imaging assessments but have limited sensitivity for characterizing disease and therapeutic response. Magnetic resonance imaging (MRI) endoscopy is a newer idea employing tiny catheter-mounted detectors connected to the MRI scanner. It can see through vessel walls and provide soft-tissue sensitivity, but its slow imaging speed limits practical applications. Our goal is high-resolution MRI endoscopy with real-time imaging speeds comparable to existing modalities. *Methods*. Intravascular (3 mm) transmit-receive MRI endoscopes were fabricated for highly undersampled radial-projection MRI in a clinical 3-tesla MRI scanner. Iterative nonlinear reconstruction was accelerated using graphics processor units connected via a single ethernet cable to achieve true real-time endoscopy visualization at the scanner. MRI endoscopy was performed at 6-10 frames/sec and 200-300 *μ*m resolution in human arterial specimens and porcine vessels *ex vivo* and *in vivo* and compared with fully sampled 0.3 frames/sec and three-dimensional reference scans using mutual information (MI) and structural similarity (3-SSIM) indices. *Results*. High-speed MRI endoscopy at 6-10 frames/sec was consistent with fully sampled MRI endoscopy and histology, with feasibility demonstrated *in vivo* in a large animal model. A 20-30-fold speed-up vs. 0.3 frames/sec reference scans came at a cost of ~7% in MI and ~45% in 3-SSIM, with reduced motion sensitivity. *Conclusion*. High-resolution MRI endoscopy can now be performed at frame rates comparable to those of X-ray and optical endoscopy and could provide an alternative to existing modalities, with MRI’s advantages of soft-tissue sensitivity and lack of ionizing radiation.

## 1. Introduction

Atherosclerosis is a prevalent factor in cardiovascular disease and a leading cause of mortality and morbidity [1-3]. While over a million X-ray-guided catheterizations are performed in the USA annually to diagnose and treat the disease, X-ray angiography can only detect lumen contours which limit its ability to assess early and advanced lesions and their progression [4-6]. Coronary X-ray computed tomography (CT) angiography can assess plaque burden via the presence of calcification but is unable to characterize many of the soft-tissue pathologies that distinguish the American Heart Association’s (AHA) classifications for vessel disease [7, 8]. Other minimally invasive imaging options include intravascular (IV) ultrasound (IVUS) and optical coherence tomography (OCT), which are clinically available but not widely used. These can offer improved spatial resolution and contrast for evaluating stenoses, identifying potentially vulnerable lesions, and facilitating intervention [9-11]. Confounding factors are the presence of calcifications in the case of IVUS [12, 13] and optical penetration and a requirement for blood-free access to the vessel wall in the case of OCT [14, 15]. Moreover, all of these modalities employ X-ray guidance and hence expose patients and operators to ionizing radiation.

IV magnetic resonance imaging (MRI) is a newer approach that employs miniature MRI detector coils mounted on guidewires or catheters for use in higher-field MRI scanners. It can provide high-resolution and soft-tissue contrast for characterizing different stages of vessel disease without using X-rays [16-18]. An “MRI endoscopy” mode is also possible with IV MRI, wherein a continuous stream of images is acquired from the point-of-view of the detector coil, to which the image frame is intrinsically locked. This is achieved by dispensing with the traditional MRI slice-selective excitation which is fixed to the scanner’s frame of reference. Instead, the highly localized receiver sensitivity profile of the tiny MRI coil, combined with an endoscopic MRI sequence that employs adiabatic MRI radiofrequency (RF) excitation, limits MRI sensitivity to a 3-5 mm (full-width-half-maximum) thick uniformly excited “sensitive disk” which is intrinsically locked to the coil and moves with it. Like an optical endoscope used for colonoscopy, the MRI endoscope provides a “probe’s eye” view from inside the vessel. Unlike optical endoscopy, MRI endoscopy can see through the vessel contents and the vessel wall to potentially locate and characterize transluminal and extravascular disease as the probe is advanced [17, 19]. Unfortunately, to date, MRI endoscopy has been limited to about 2 frames per second (fps) at 300 *μ*m resolution for real-time visualization in blood vessels [17]. The speed is limited because state-of-the-art sensitivity encoding (SENSE) MRI methods are not possible with a single-channel endoscope and because the reconstruction (and acceleration) rates of existing iterative compressed MRI methods have not been fast enough and have required off-line iterative processing that is unsuitable for practical endoscopy applications [20]. Thus, true real-time MRI endoscopy at frame rates suitable for interventional, catheterization, or endoscopy procedures has not yet been realized.

**Figure 1:**
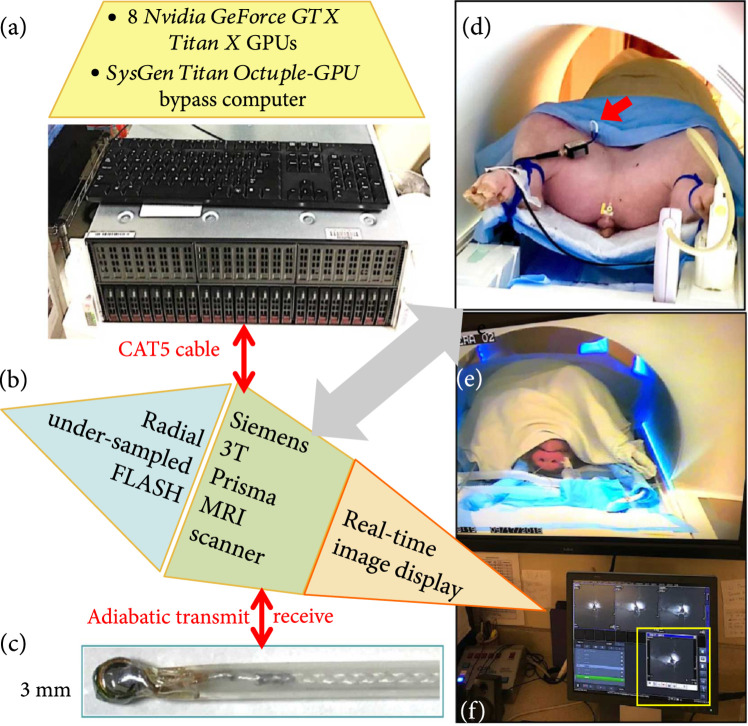
(a-c) Block diagram of the MRI endoscope. (a) Eight cascaded GPUs controlled locally by a GPU computer programmed to perform undersampled radial image reconstruction in real time are connected to (b), a 3 T clinical MRI scanner via a CAT5 computer cable (red arrow). The MRI scanner is programmed for high-speed (fast low-angle shot, FLASH) MRI to drive (c), a 3 mm transmit/receive endoscope (red arrow), via a tuned interface and preamplifier. (d) The endoscope accesses the vasculature of a (porcine) subject lying in the magnet of the MRI scanner (grey arrow) via the femoral artery, during (e) video monitoring as (f) the endoscopic images are displayed in real time on the scanner’s display window (yellow box) and on an in-room monitor.

A highly accelerated real-time MRI system was recently developed for conventional multichannel MRI with transmitters and receivers that are fixed to the scanner frame of reference and its localizing gradient system [21-23]. The system uses highly undersampled radial pulse sequences and a temporally regularized, iterative, nonlinear inversion (NLINV) reconstruction algorithm, implemented with cascaded graphics processing units (GPUs) to provide essentially instantaneous image reconstruction and visualization. Here, we report the novel incorporation of this technology to create a real-time (single-channel) MRI endoscope that can move relative to the scanner’s frame of reference with continuous microscopic visualization at up to 10 fps, in-plane resolution of 200-300 *μ*m, and an imaging field-of-view (FOV) of 2-3 cm without inducing significant local heating. After calibration and safety testing, a uniform MRI excitation flip angle (FA) is excited by the tiny coil at the end of the endoscope using adiabatic “BIR-4” RF pulses [24], and the size of the FOV or “sensitive disk” is adjusted independently of the scanner’s frame of reference. The software operates on a regular clinical MRI scanner with the GPU hardware connected via a high-speed ethernet cable (Figure 1). Results from animal and human vessels *ex vivo* and from animal vessels *in vivo* are demonstrated, and the structural and temporal information in the highly accelerated image streams is compared with fully sampled MRI endoscopy scans using image similarity metrics and with tissue dissections and histology.

## 2. Results

**Figure 2:**
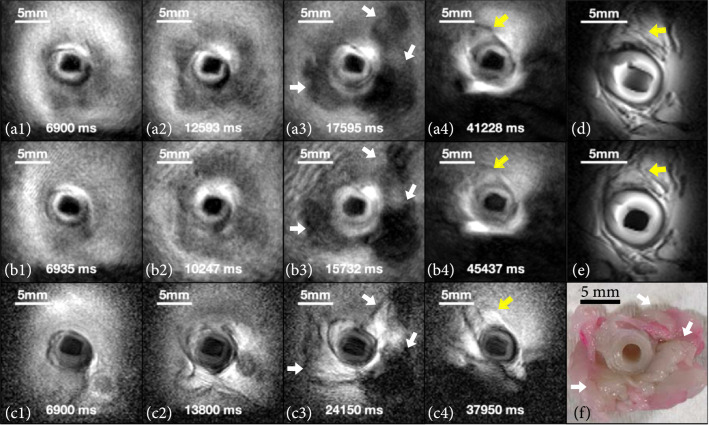
MRI endoscopy of a porcine carotid artery *ex vivo*. Imaging frames at 6 fps (a1-a4) and 10 fps (b1-b4) and from fully sampled 0.3 fps MRI endoscopy (c1-c4) acquired at approximately the same locations. Videos are provided in Supplementary Materials. (d, e) High-resolution “static” 3D endoscopic images at the location of the 4^th^ column. (f) Dissection at the location of the 3^rd^ column (white arrows: adipose; yellow arrows: attached tissues).

Figure 2 shows endoscopic image frames from a pig carotid artery specimen in a 0.35% saline phantom at 200 *μ*m nominal (in-plane) resolution. Frames in rows (a1-a4) are recorded using highly undersampled radial acquisitions at five different positions at 6 fps and visualized in real time. Frames (b1-b4) are recorded in real time from approximately the same locations but at 10 fps. These are, respectively, 20 and 33 times faster than 0.3 fps 200 *μ*m resolution images acquired using a “conventional” fully sampled endoscopy sequence depicted in frames (c1-c4). Videos of the image streams are presented in Supplementary Materials. Figures 2(d) and 2(e) show a “static” fully sampled three-dimensional (3D) endoscopic image acquired in 368 s at the same nominal resolution as a reference. Figure 2(f) shows a vessel dissection.

**Figure 3:**
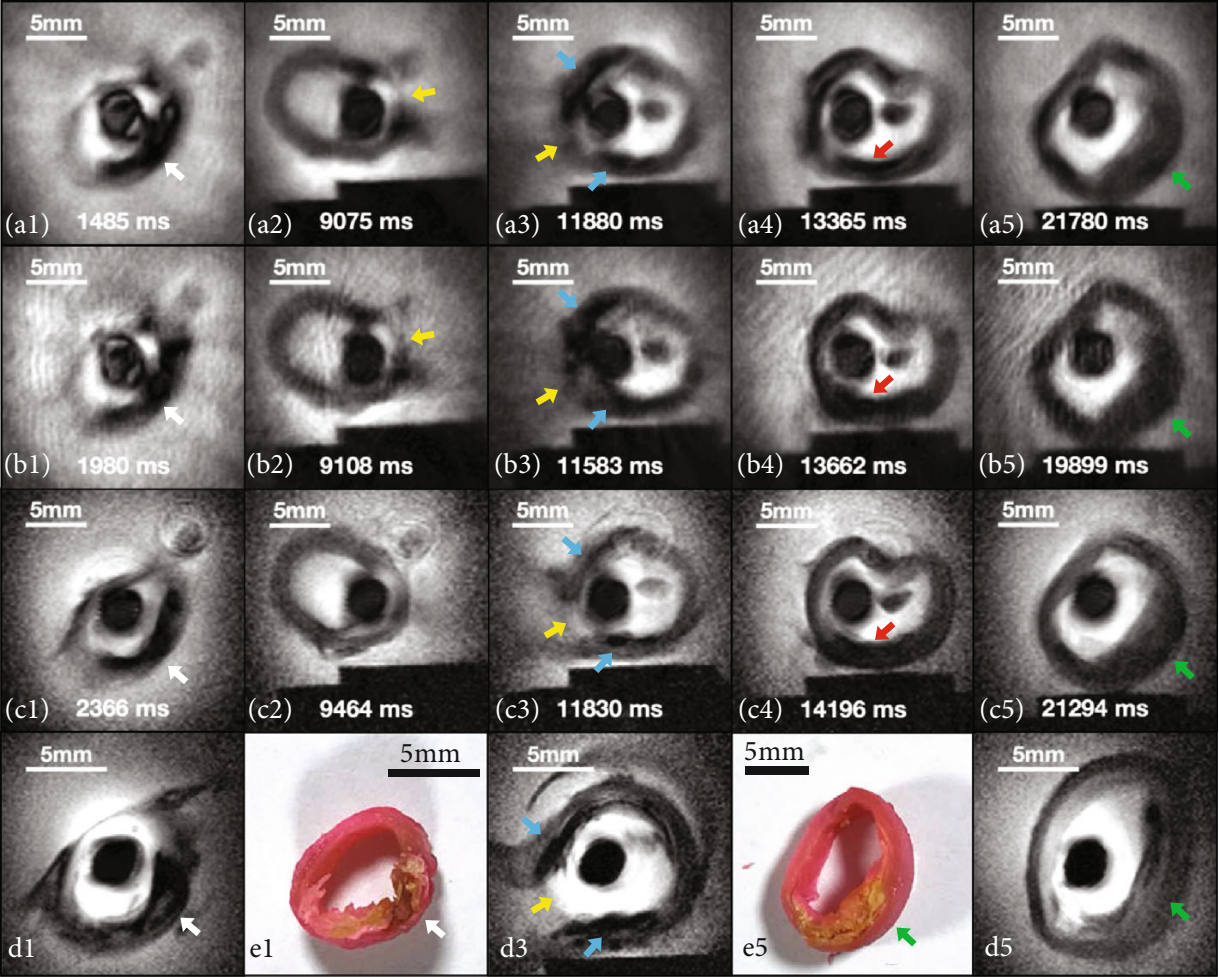
MRI endoscopy of a diseased ex vivo human iliac artery. Image frames from 6 fps (row a) and 10 fps (row b) real-time scans, an 0.3 fps fully sampled endoscopy stream (row c) and high-resolution static 3D endoscopic reference scans (row d) at approximately the same locations transitioning from a branch to the main lumen (see Supplementary Material Figure S2). The vessel wall opens at the transition (yellow arrows in (a2, a3, b2, b3, c3, and d3)). Videos are provided in Supplementary Materials. Each column of images corresponds to approximately the same locations except the vessel wall dissection in (e1) corresponding to (d1) and (e5) corresponding to (d5). Fibrocalcific plaque (white, blue, red, and green arrows) and intimal thickening (green arrow) were identified by Movat staining on histology of the sections.

Figure 3 presents images from a diseased human iliac artery specimen obtained from our institution’s autopsy service, immersed in saline. Each column derives from approximately the same locations. Frames in row (a) and row (b) were sequentially acquired in real time using highly undersampled 220 *μ*m endoscopy sequences, respectively, at 6 fps and 10 fps. As the endoscope is withdrawn through the artery, the branching of the internal iliac is seen as a lateral opening in the vessel wall (Figures 2(a2), 2(a3), 2(b2), and 2(b3); yellow arrows). Videos of these image streams plus a 0.3 fps, 220 *μ*m “conventional” fully sampled endoscopy stream 20-33 times slower are included in Supplementary Materials. Images in row (c) are reference scans from fully sampled 0.3 fps endoscopy stream, and those in row (d) derive from high-resolution (nominally 100 *μ*m) static 3D endoscopic images acquired in 112 s. Poststudy dissections of the vessel wall in (e1) and (e5) show atherosclerosis with fibrocalcific plaques and intimal thickening corresponding to images (d1) and (d5), respectively.

**Figure 4:**
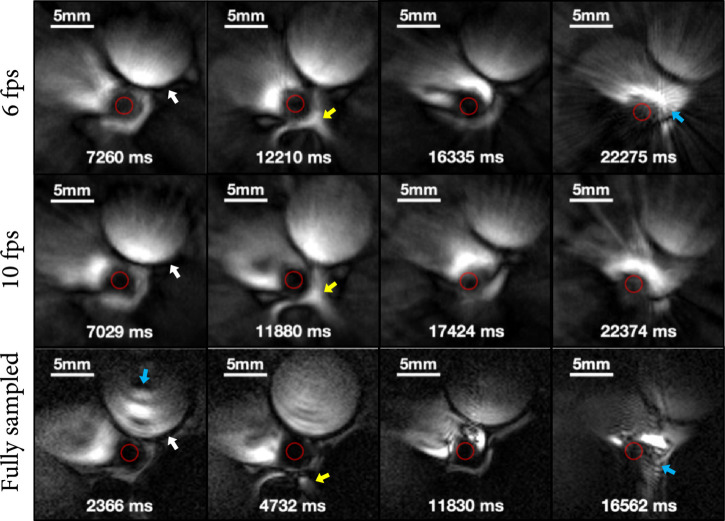
MRI endoscopy frames from a porcine IVC in vivo. Rows 1-3 were acquired at 6 fps, 10 fps, and fully sampled at 0.3 fps. Images in each column are in approximately the same location (white arrows: the aorta abutting the IVC; yellow arrows: structures near the spine; red circles: coil positions; blue arrows: motion artifact in the slower scans).

*In vivo* MRI endoscopy frames from a pig inferior *vena cava* (IVC) are shown in Figure 4: the endoscopy streams are included in Supplementary Materials as well. The top two rows of images were acquired using undersampled 6 fps and 10 fps endoscopy, respectively, 20 and 33 times the speed of the fully sampled 0.3 fps images shown in the third row. The nominal spatial resolution was 220 *μ*m, and each column of images is from approximately the same location for comparison.

The mutual information (MI) and the three-component weighted structural similarity index measure (3-SSIM) were used to compare the similarity between the high-speed 6-10 fps (200-300 *μ*m nominal resolution) highly undersampled images and the slow “conventional” fully sampled scans. MI is widely used for registering images from the same source that have different contrasts such as medical images acquired with different modalities [25], or as here, 3D “static” reference scans acquired with different scan protocols. Table 1 summarizes the results from 11 samples (4 porcine vessels and 7 human diseased vessels) acquired with 3D MRI (100-200 *μ*m nominal resolution; 50-370 s scan time) and fully sampled endoscopy at 0.3 fps (200-300 *μ*m nominal resolution). Two-way ANOVA showed significant differences in MI between the high-resolution static 3D reference scans acquired in 50-370 s which used Cartesian spatial encoding and the 0.3-10 fps dynamic scans acquired with radial encoding (p<0.001). However, while the fully sampled 0.3 fps method outperformed the 6-10 fps highly undersampled scans (p<0.001), the 20 to 33-fold acceleration incurred only an ~7% cost to MI. The 3-SSIM metric, which reflects degradation in image contrast and emphasizes structural errors of perceptual significance [26, 27], was only slightly better at 6 fps than at 10 fps (57 vs. 55; Table 1) using the 0.3 fps scans for reference. These results suggest little loss of fidelity at the higher scan rate.

**Table 1:** Comparison of image similarity indices.

Metric	0.3 fps	6 fps	10 fps
MI (vs. 3D 50-370 s reference)	25.8±4.1	$23.8\pm 2.9^{*}$	$24.2\pm 3.1^{*}$ ^†^
3-SSIM vs. 0.3 fps	—	57.3±10.4	55.1±9.7 ^§^
3-SSIM vs. 6 fps	—	—	68.0±10.8
ΔL (mm)	—	0.34±0.20	0.28±0.14 ^#^

Values are $\text{means}\pm \text{standard}\;\text{deviations}\;\left(\text{SD}\right)$. MI and 3-SSIM are in %. ${}^{*}p<0.001$ vs. 0.3 fps scans (two-way ANOVA, degrees of freedom = 2). ^†^Not significantly reduced vs. 6 fps scans. ^§^$p<10^{-5}$ vs. 6 fps scans (paired t-test). ^#^p=0.03 vs. 6 fps (paired t-test).

As a gauge of motion sensitivity, a comparison of the average frame-to-frame displacement of the endoscope, ΔL, showed an 18% reduction at 10 fps vs. 6 fps (p=0.03). The apparent image signal-to-noise ratio (SNR) in a 1 cm square centered on the endoscopy coil was 15±3 averaged from the 11 real-time endoscopy samples recorded at 6 fps and at 10 fps.

## 3. Discussion

This work demonstrates for the first time that the speed of MRI endoscopy can be increased twenty- to thirtyfold to 10 fps and visualized in real time on a clinical 3 T MRI scanner using high-speed highly undersampled MRI radial acquisition, highly parallelized GPU-based reconstruction, and a transmit/receive device moving independently of the scanner frame of reference. Undersampling was previously proposed as a means of accelerating MRI endoscopy [20]. However, it was only shown on retroactively acquired, fully sampled data, and the iterative reconstructions took minutes to complete, resulting in a process that was incompatible with any practical endoscopy application. The present results are five times faster than prior best efforts at real-time endoscopy [17]. The advance here was achieved using stand-alone hardware developed for real-time MRI that is connected to the scanner via a simple ethernet cable [21] and a modified MRI pulse sequence that replaced slice selection with adiabatic excitation to provide imaging from the viewpoint of the coil at the endoscope’s tip. The MRI frame rates of 6-10 fps are comparable to X-ray fluoroscopy rates of 7.5-15 fps and are faster than a 4 fps fluoroscopy rate recently advised for mitigating radiation dose [28] which is not an issue for MRI. Our MRI endoscopy frame rates are also comparable to the 2-10 fps rates used in optical gastrointestinal endoscopy procedures [29, 30]. MRI endoscopy enabled continuous real-time feedback to guide catheter passage through blood vessels *ex* and *in vivo*. It could thus serve as a value-added complement or alternative to diagnostic or interventional X-ray catheterization, optical endoscopy, and IVUS procedures, benefitting from MRI’s advantages of soft-tissue sensitivity, lack of ionizing radiation, and the ability to see through vessel contents and vessel wall uncompromised by calcifications, as compared to optical and IVUS methods.

Highly accelerated, undersampled, real-time MRI endoscopy is not artefact- or cost-free, however. Our analysis showed that highly undersampled real-time MRI endoscopy can achieve ≥20 times the frame rate of conventional fully sampled endoscopy at a cost of ~7% reduction in mutual information (MI). Compared to the fully sampled experiments, there was no significant difference in MI between 6 and 10 fps. The small difference in the 3-SSIM metric for the 10 fps and the 6 fps data streams, as compared to the 0.3 fps scans (Table 1), is likely attributable to an increase in undersampling “spoke” artifacts at the higher speed (compare Figures 2(b) and 3(b) with Figures 2(a) and 3(a)). Nevertheless, 10 fps provided smoother frame transitions and less sensitivity to motion than 6 fps, with a reduction of about 18% in the frame-to-frame displacement (ΔL) to a value (0.28 mm) commensurate with the spatial resolution and comparable to the increase in speed. Note that spoke artefacts were attenuated and conspicuity improved in the peripheral FOV using a simple analytical preprocessing filter applied to the raw projection data, which better compensated for the extreme inverse-radial (1/r) inhomogeneity of the endoscopic coil and downstream NLINV reconstruction (Supplementary Materials). Also, while the comparative metrics were averaged from 11 sets of cine streams, a caveat is that they all derive from images acquired at different rates and times from vessel transits that are unlikely to follow exactly the same track or sample the exact same locations due to the very nature of endoscopy. Thus, a perfect MI or 3-SSIM cannot be expected.

Surprisingly perhaps, the difference in artefacts at 6 fps versus 10 fps is less obvious *in vivo* (Figure 4) than *ex vivo* (Figure 3). This may reflect a trade-off between the improved ability to freeze physiological motion in the living animal at higher speeds, offsetting the increase in spoke artefacts at the higher frame rate. Still, moving the probe too fast or nonuniformly within the frame acquisition period (jerking or changes in catheter-sheath friction, etc.) is liable to cause “glitches” of intense spoke artifacts from the afflicted projections (annotated in the supplementary videos). The effect is not uncommon to other intravascular imaging modalities wherein glitches are ignored or the frames automatically dropped from the imaging stream. Nevertheless, there is certainly room to improve artifact suppression. Indeed, the hyperintense radial artifacts resemble metallic artifacts in X-ray CT images which have been addressed using data-adaptive artifact reduction algorithms [31, 32] that might be adaptable here. Meanwhile, given the real-time feedback at our frame rates, the MRI operator can just pause in advancing the endoscope, whereupon replacement images will appear in very short order. By comparison, the much slower speed of fully sampled MRI endoscopy renders it prone to artifacts from motion occurring during the acquisition of each image frame, even when the probe is not being advanced (Figure 4). When motion is paused at locations of interest, more projections can always be acquired at high speed to improve spatial—at the expense of temporal—resolution. A regular high-speed mode can be resumed as soon as the probe is advanced further or retracted.

**Figure 5:**
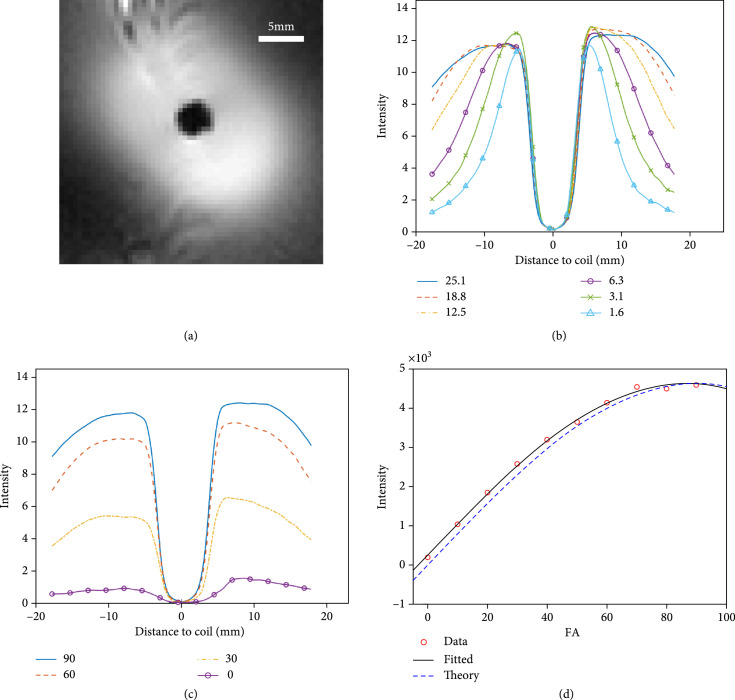
Adjusting the adiabatic (BIR-4) excitation pulse during endoscopic MRI to independently control field-of-view (FOV) and sensitivity. (a) Image of the endoscope’s “sensitive disk” in a homogeneous phantom (gradient refocused echo, GRE image; repetition period, TR=5 s; echo time, TE=4.36 ms; nominal BIR-4 flip angle, $\text{FA}=90^{{}^{\circ}}$; field-of-view, FOV=38 mm; and transmit voltage, Tx=25.1 V). (b) Adjusting FOV. Reducing pulse power (Tx) at a constant $\text{FA}=90^{{}^{\circ}}$ decreases the BIR-4 pulse’s threshold for adiabaticity and hence the FOV. (c) Sensitivity optimization. The average radial MRI signal plotted with different BIR-4 FAs at Tx=25.1 V. The FOV is independent of FA. (d) Calibration of FA. The image intensity as a function of the nominal FA of the BIR-4 pulse fitted to a sine function reveals a fixed 5° offset error in FA.

A characteristic of MRI endoscopy is the highly nonuniform excitation field ($B_{1}$) around the endoscopic coil when it is used for transmission and—by the principle of reciprocity—its highly nonuniform sensitivity when deployed as a receiver (Figure 5). Both of these properties are used to localize the endoscopy coil first by enabling the elimination of MRI slice selection, which is normally fixed to the scanner frame of reference, and second by reducing the effective FOV and hence the number of spatial encoding steps or projections needed for imaging. The adiabatic (BIR-4) pulse maintains a uniform MRI flip angle (FA) inside this effective FOV, which may be defined as the volume of tissue (“sensitive disk”; Figure 5(a)) enclosed by the $B_{1}$-contour that corresponds to the pulse’s threshold for adiabaticity. Outside of this $B_{1}$-threshold, the FA fades rapidly to zero. MRI endoscopy requires two independent adjustments of the pulse. First, the $B_{1}$ amplitude sets the spatial extent of the sensitive disk (Figure 5(b)), and second, the pulse’s internal phase is adjusted to set FA within the FOV to optimize the SNR and the desired image contrast (Figure 5(c)). Regarding the latter, the use of the endoscopy coil for excitation, the use of adiabatic pulses to combat the nonuniform $B_{1}$, and the focus on speed to facilitate MRI endoscopy applications analogous to those of other modalities are all confounding factors for adjusting MRI contrast by conventional means while the endoscope is moving. The efficacy of adding a $\text{FA}=180^{{}^{\circ}}$ pulse to generate MRI relaxation-time ($T_{1}$) contrast, for example, depends on the speed that the probe advances to locations outside the range of the 180° pulse. With the probe stationary, however, MRI relaxation times *can* certainly be quantified using conventional or accelerated approaches [18].

The primary safety concern for internal devices is the localized heating induced by MRI’s radiofrequency (RF) pulses, which is not well gauged by the volume-averaged RF-specific absorption rates used for general MRI [33, 34]. With an FOV of ~20 mm (Figure 5(b); Tx≤7.8 V), local tissue heating during endoscopy at the stated scan rates was ≤0.5°C, in accordance with prior studies showing no significant heating or thermal injury [16, 17]. When the FOV was increased to ~30 mm by increasing $B_{1}$ amplitude (Tx=18.8 V), a local temperature rise of 3.5°C was recorded after continuously streaming 600 images from a single location. However, heating at the higher power level is reduced with the endoscope unfixed and moving through a local perfused volume such as a blood vessel. The maximum temperature rise is localized in the immediate vicinity of the coil [16, 17] and can also be monitored by high-resolution MRI thermometry performed using the endoscope [35, 36].

In conclusion, high-speed MRI endoscopy, unlocked from the scanner frame of reference, can be combined with undersampling and real-time image reconstruction technologies to match the speed of existing clinical catheterization and endoscopy procedures and could provide a useful complement to established minimally invasive imaging modalities including X-ray fluoroscopy, IVUS, OCT, and optical endoscopy [37]. It has the potential advantages of multifunctional imaging afforded by MRI, including thermal imaging for monitoring transvascular [36] and perivascular ablation therapies [35] and parametric imaging for classifying vessel disease as demonstrated previously [18]. The main hurdles to the technology are primarily those associated with interventional MRI in general: its expense, the limited availability of scanners in interventional settings, and accessibility to patients, interventionalists, recovery facilities, and expertise in interventional MRI.

## 4. Methods

### 4.1. MRI Endoscopy

Four 3 mm diameter prototype MRI endoscopes were fabricated for use in studies performed on a clinical 3 T MRI scanner (*Prisma*, Siemens Healthineers AG, Munich, Germany). The endoscopes had 3-5 turn transmit/receive coils that were tuned to the 123.2 MHz MRI frequency with microchip capacitors and mounted on 1/4-wavelength (42 cm), 50 *Ω*, flexible, 1.25 mm diameter, silver-plated microcable and enclosed in a 2.4 mm polymer sheath (Nylon 12, 64 Shore D hardness; Figure 1(c) and Supplementary Materials Fig. S2). The endoscopes were matched to 50 *Ω* when immersed in saline with tissue-comparable RF electrical properties (Supplementary Material Fig. S2) and interfaced to the MRI scanner via two ganged (gain, 26 dB+20 dB) low-noise (noise figure, 0.4-0.5 dB) preamplifiers at the receiver’s front-end [16]. A switchable 20 dB attenuator was connected to the scanner’s RF transmitter amplifier output.

The MRI acceleration unit was comprised of 8 *Nvidia GeForce GTX Titan X* (*Nvidia*, Santa Clara, CA) GPUs and a “bypass” computer (sysGen/TITAN Octuple-GPU, Sysgen, Bremen) described previously [21-23]. It was connected to the Siemens scanner via a single ethernet cable and configured to provide continuous real-time image display at the scanner console (Figure 1). MRI was accelerated using highly undersampled radial acquisitions (9-17 projections per image frame) and reconstructed in the bypass computer with a highly parallelized version of the NLINV algorithm [38] which jointly estimates image and coil sensitivity, with a minimum latency period of one frame. Although the NLINV reconstruction also accommodates corrections for the slow-varying sensitivity profiles of regular MRI coils, a spatial prefilter was applied to the raw projection data to compensate for the extreme inverse-radial (~1/r) dependence of the coil’s detection sensitivity and enhance conspicuity in the periphery of the FOV, analogous to that used previously [16]. The filter and its effect are detailed in Supplementary Materials (S1). During high-speed endoscopy, radial projections were acquired from a continuous application of a fast low-angle shot (FLASH) MRI sequence [39] programmed with adiabatic BIR-4 pulses (pulse duration: 4 ms; frequency sweep: ±10 kHz) in lieu of spatially selective excitation. Applying these pulses with the endoscopic coil as a transmitter restricted the MRI sensitivity to an approximately discoidal volume (Figure 5(a)) that moved with the probe, no longer locked to the frame of reference of the scanner’s localizing gradient system [17].

The sequence operating parameters were adjusted to match the probe’s excitation and detection sensitivity, which decline rapidly with distance from the coil. The amplitude of the excitation field ($B_{1}$) sets the adiabatic threshold of the BIR-4 pulses and effectively sets the radius of the sensitive disk. This was calibrated by varying the scanner’s nominal transmit voltage (Tx at the RF power amplifier, excluding intervening losses) from 1.6 to 25 V with the endoscope placed in the saline phantom using a long repetition period (TR=5 s) short-echo-time (TE=4.4 ms) gradient-recalled echo (GRE) BIR-4 sequence. The average radial sensitivity profile was plotted as a function of distance from the coil (Figures 5(a) and 5(b)). Nominal transmit RF voltages of 6.3 V and 18.8 V were chosen for *ex* and *in vivo* studies, to provide effective FOVs of ≥20 mm and ≥30 mm, respectively (Figure 5(b)). RF heating of the endoscope during continuous high-speed endoscopy with the probe at the same location for 2 minutes (600 images) at TR=20 ms was measured in the saline phantom in the scanner with fiber optic temperature probes placed at the tip where peak heating occurs, and at more remote locations [16, 17, 36].

The MRI FA of the BIR-4 pulse was adjusted to maximize the SNR with the probe stationary. FA is set by phase jumps within the pulse [16, 24] which are typically misset in MRI scanner hardware [40]. This is normally remedied by cycling the BIR-4 pulse’s phase which was not possible here because the extreme acceleration precluded repetition of phase-cycled projections. Instead, the FA of the BIR-4 pulses was calibrated in the saline phantom, with nominal “scanner” FAs varied between 0° and 90° at 10° increments at long TR and a fixed Tx=25.1 V (Figure 5(c)). The maximum image intensity for a given Tx was fitted to the sine of the nominal FA (Figure 5(d)) to determine the true FA [40], and the resultant offset error (5°) subtracted from the scanner FA when applying our BIR-4 sequences and reporting the FAs.

### 4.2. Ex Vivo Experiments

High-speed real-time MRI endoscopy was first tested in healthy porcine blood vessels (4 samples) and in diseased human blood vessels (7 samples) *ex vivo*. Fresh porcine vessels (≤24 hrs post mortem) were obtained from commercial research suppliers (Animal Biotech Industries, Doylestown, PA; Spear Products Inc., Coopersburg, PA). Deidentified human vessels harvested postmortem from elderly voluntary donors were obtained from this institution’s autopsy service. These commonly show evidence of atherosclerotic lesions and calcifications detectable by MRI endoscopy. Vessels were mounted on a rubber platform at the center of a (0.35%) saline solution placed in the MRI scanner. The MRI endoscope was placed at the distal end of the vessel, the endoscopy sequence commenced, and the coil retracted by an operator from the end of the scanner (Supplementary Fig. S2).

Real-time BIR-4 FLASH MRI endoscopy of porcine carotid arteries was performed at 6 and 10 fps (FOV=45 mm; resolution=200 *μ*m; $\text{FA}=10^{{}^{\circ}}$; TR/TE=11.5/6.68 ms; projections/image frame=15 for 6 fps (172.5 ms/frame), 9 for 10 fps (103.5 ms/frame); and pullback rate≈2 mm/sec). A fully sampled BIR-4 GRE Cartesian MRI sequence used previously [16, 17] (denoted as “conventional” MRI endoscopy) was applied at 0.3 fps to provide an unaccelerated reference for comparison (FOV=45 mm; resolution=200 *μ*m; $\text{FA}=10^{{}^{\circ}}$; TR/TE=15/6.61 ms; and 3.34 s/frame). The duration of each endoscopy stream was ~30 s. 3D high-resolution “static” images were also acquired at locations of interest (BIR-4 GRE; FOV=45 mm; resolution=200 *μ*m; phase encoding steps, PE=225; $\text{FA}=10^{{}^{\circ}}$; TR/TE=100/6.61 ms; SL=2 mm; 16 slices; and scan time=6.1 min).

Human iliac artery segments were studied with real-time MRI endoscopy at 6 fps and 10 fps (FOV=40 mm; resolution=220 *μ*m; $\text{FA}=10^{{}^{\circ}}$; TR/TE=11/6 ms; projections/image frame=15 for 6 fps (165 ms/frame), or 9 for 10 fps (99 ms/frame); and pullback rate≈4 mm/sec) and with fully sampled conventional MRI endoscopy (FOV=40 mm; resolution=220 *μ*m; PE=182; $\text{FA}=10^{{}^{\circ}}$; TR/TE=13/5.55 ms; and 2.37 s/frame) for comparison. The locations of suspected vessel lesions were identified, and static 3D high-resolution MRI was then performed at these locations (BIR-4 GRE; FOV=50 mm; resolution=100 *μ*m; $\text{FA}=10^{{}^{\circ}}$; TR=20 ms; TE=10.1 ms; SL=2 mm; 16 slices; and scan time=1.9 min). The vessels were then removed and dissected at image locations, fixed, and Movat-stained for histology.

### 4.3. *In Vivo* Experiments

*In vivo* studies were performed with the MRI endoscope in the IVC of anaesthetized 50 kg female Yorkshire pigs (Figures 1(d) and 1(e)). The IVC was accessed percutaneously via the femoral vein under X-ray fluoroscopy guidance in studies approved by our Institutional Animal Care and Use Committee. During MRI endoscopy, an operator at the MRI scanner bore-opening retracted the endoscope continuously as the image stream was displayed in real-time on the console monitor (Figure 1(f)). A portable in-room console was available for monitoring or adjusting sequences. IVC endoscopy was performed at 300 *μ*m resolution using the 6 and 10 fps accelerated sequences (FOV=40 mm; resolution=220 *μ*m; $\text{FA}=10^{{}^{\circ}}$; TR/TE=11/6 ms; projections/image frame=15 for 6 fps (165 ms/frame), or 9 for 10 fps (99 ms/frame); and pullback rate≈4 mm/sec) and with the 0.3 fps fully sampled “conventional” sequence (FOV=40 mm; resolution=220 *μ*m; PE=182; $\text{FA}=10^{{}^{\circ}}$; TR/TE=13/5.55 ms; and 2.37 s/frame).

### 4.4. Image Evaluation

To correlate dynamic endoscopy series acquired at 0.3 fps, 6 fps, and 10 fps with static 3D reference scans acquired from the same samples, the MI (normalized with range [0, 1]) [25] of each dynamic frame was determined for each static (3D) reference image slice, and the dynamic frame with the largest MI assigned to that reference location (see Supplementary Materials S3 (available here)). The average MI for each dynamic endoscopy series at each frame rate as compared to the reference scans was then computed for each series. The structural similarity of real-time endoscopic images acquired at 6 fps and 10 fps was compared with the 0.3 fps conventional endoscopy images by first registering them based on the maximum MI as above. The average 3-SSIM (range, [0, 1]) [26, 27] was then computed. The 3-SSIM of the 10 fps vs. the 6 fps scans was calculated similarly. Potential sensitivity to motion was compared by computing the average ΔL between adjacent frames of the 6 and 10 fps data streams (Equation A2, Supplementary Material S3).

MI, 3-SSIM, and ΔL were determined for 11 samples (4 porcine vessels and 7 diseased human vessels) from which 3 dynamic scans (0.3 fps, 6 fps, and 10 fps) and one static (3D) reference scan were acquired. MI was compared using two-way analysis of variance (two-way ANOVA) with frame rates and samples as independent variables. Multiple comparisons of different frame rates were performed using Tukey’s honest significance test. Differences between 3-SSIM at 0.3 fps and ΔL at 6 and 10 fps were assessed by paired t-testing. The apparent SNR was calculated in the accelerated scans from the quotient of the average signal in a 1 cm square centered on the endoscope and the SD of background noise measured in a 0.5 cm semicircle 1.5 cm from the center.

## Data Availability

De-identified DICOM format files of endoscopic image streams are available upon application to the corresponding author.

## Supplementary Materials

Supplementary Materials S1: preprocessing filter. S2: illustration of MRI endoscopy. S3: image comparison metrics. S4: MRI endoscopy videos.
