# Supplementary material for: Real-Time High-Resolution MRI Endoscopy at up to 10 Frames per Second
Source: BME Front. 2021 Feb 17;2021:6185616. doi: 10.34133/2021/6185616 (PMC10521714; doi:10.34133/2021/6185616)
Supplement: Supplementary Materials — S1: preprocessing filter. S2: illustration of MRI endoscopy. S3: image comparison metrics. S4: MRI endoscopy videos. [file 6185616.f1.zip › BMEF-D-20-00017.Supplement.R1.pdf]

Supplementary Material: "Real-time high-resolution MRI Endoscopy at up to 10 Frames per Second", Liu et al

S1. Preprocessing Filter

A simple inverse radial spatial filter was used in prior work to compensate for the approximately inverse-radial (1/r) coil sensitivity profile of endoscopic MRI detectors[16]. Here, the NLINV reconstruction removes most of the undersampling artifacts[21]. without such correction. However, the highly nonuniform sensitivity profile can suppress some of the useful information in the peripheral FOV during downstream reconstruction. We found that preprocessing the projection data to provide a slower-varying coil sensitivity profile improves image homogeneity, contrast and conspicuity. As the raw (inverse Fourier-transformed) data is an integral of the signal along the projection path, a heuristic preprocessing filter was designed to support the underlying solenoidal detection sensitivity of the endoscope (Fig. 1C), with the correction profile:

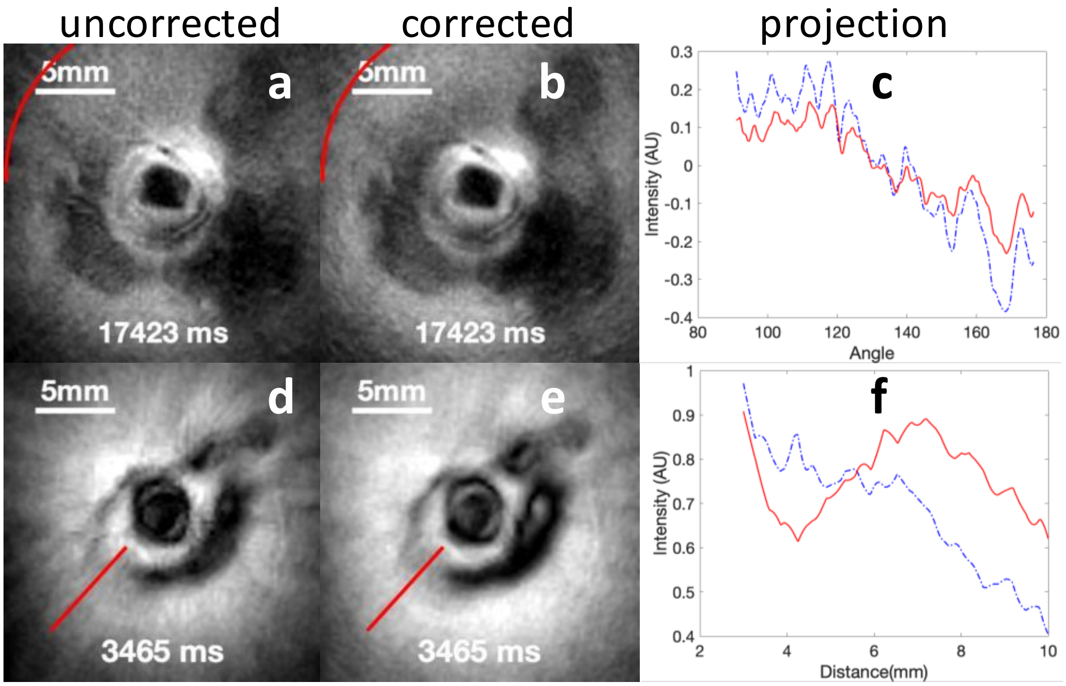

Figure S1. Example of image frames from 6fps porcine carotid (a, b) and human iliac (d,e) specimens without pre-filtering (a, d) and after applying Eq. (S1) (b, e). Part (c) plots image intensity vs. polar angle around the arcs indicated in red in the images. These plots shows a reduction in peripheral angular fluctuations associated with radial spokes in the pre-filtered image (red) vs. no filter (blue). Part (f) shows the improvement in uniformity in the radial dimension afforded by pre-filtering (red curve vs. blue curve).

$$f(r) = \int_{-d/2}^{d/2} dx \Big/ \int_{-d/2}^{d/2} \frac{1}{x^2 + r^2} dx = \frac{\sqrt{R^2 - r^2}}{\log\left|\frac{R + \sqrt{R^2 - r^2}}{r}\right|} \qquad (S1)$$

where r is the distance to the coil center, R is the coil's sensing range, and $d = 2\sqrt{R^2 - r^2}$.

The filtered projection data are fed to the GPU bypass computer and acceleration unit for reconstruction with the NLINV program. Examples of images in Figure S1 with and without the Eq. (S1) pre-filter correction, show a reduction in both the angular intensity fluctuations and the radial decline in signal sensitivity. Endoscopy videos (Supplementary Material S5) with and without filtering are included for side-by-side comparisons.

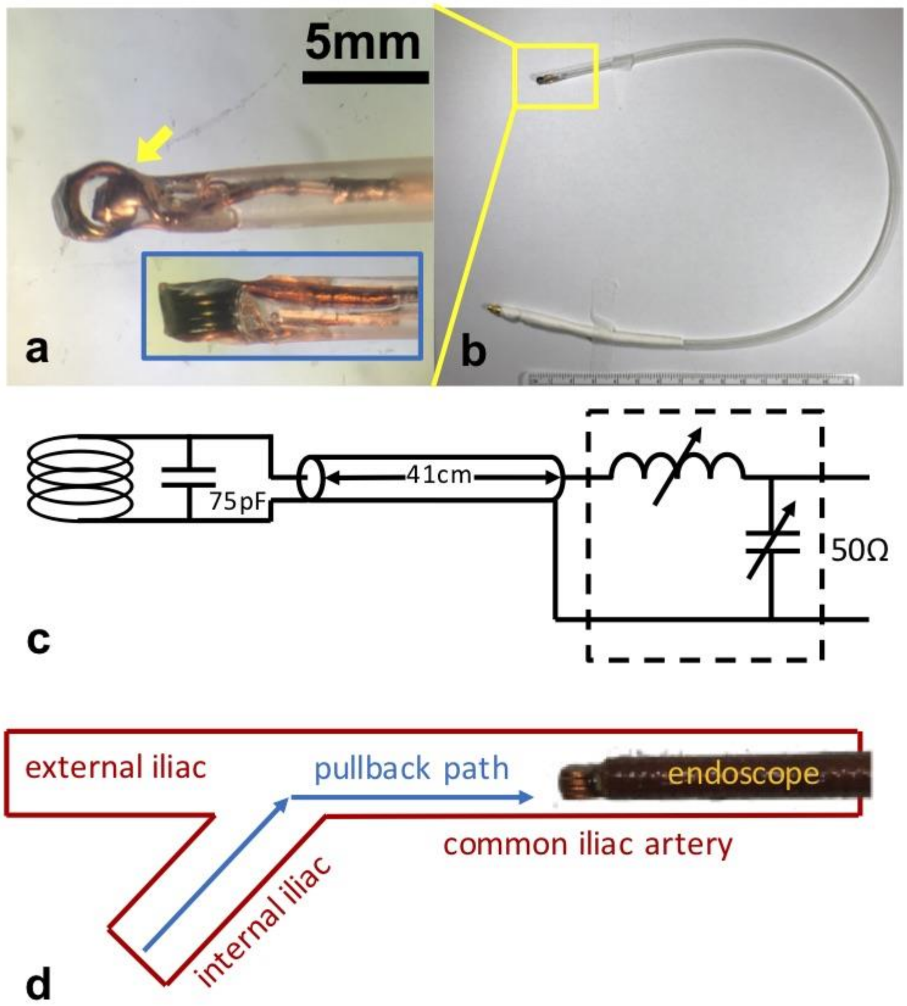

Figure S2. MRI endoscope. Photos of: (a) a 5-turn, 3mm diameter, endoscopic MRI coil tuned to the 123.2MHz MRI frequency with a tuning capacitor inside (yellow arrow). (b) The coil is mounted on a flexible 41-cm 50Ω flexible coaxial cable (silver-plated copper conductor; perfluoroalkoxy-dielectric and jacket; 1.25mm outer diameter) in a polymer sheath (Nylon 12, 64 Shore D hardness, 2.4mm outer diameter). (c) Circuit diagram showing the coil, coaxial cable and matching circuit (dashed box) which is connected to the scanner preamplifiers. (d) A 'pullback' protocol in the left common iliac artery from an internal iliac branch.

S2. Illustration of MRI Endoscopy

Illustrations of the MRI endoscopy device and experiment are shown in Figure S2.

S3. Image Comparison Metrics

1. Mutual Information and the Structural Similarity Index

Mutual information (MI) is a widely used criterion for aligning multi-modal medical images (CT, MRI, PET, etc.) describing the amount of information shared by two images of different contrast characteristics[25]. In this study, MI of two images A and B were calculated as

$$\mathrm{MI}(A,B) \;\; = \mathrm{H}(A) + \mathrm{H}(B) - \mathrm{H}(A,B)$$
$$= \sum_{a}\sum_{b} p_{AB}(a,b)\log\frac{p_{AB}(a,b)}{p_A(a)p_B(b)} = \sum_{\mathrm{a}}\sum_{\mathrm{b}} \frac{\mathrm{h_{AB}(a,b)}}{\mathrm{N}}\log\frac{\mathrm{h_{AB}(a,b)}}{\mathrm{h_A(a)h_B(b)}} + \log \mathrm{N} \tag{A1}$$

where $\mathrm{H(X)}$ is the entropy of image X; $\mathrm{p_{AB}(a,b)}$ is the joint probability distribution of pixel values; $\mathrm{p_A(a)}$ and $\mathrm{p_B(b)}$ are marginal probability distributions; $\mathrm{h_A B(a,b)}$ is the joint histogram; $\mathrm{h_A(a)}$ and $\mathrm{h_B(b)}$ are histograms of each image; N is the number of pixels in each image. The metric normalized for the number of pixels is

$$\frac{2MI(A,B)}{H(A)+H(B)} \in [0,1]$$

The structural similarity index measure (SSIM) is a perception-based measure of similarity of two images for quantifying quality degradation[26]. The three-component weighted SSIM (3-SSIM) better correlates with perception by emphasizing edge regions of features vs. smooth regions[27]. This is better-suited to endoscopy where most information is concentrated within the focus vs. the large uniform area around the periphery. In the present study 3-SSIM was calculated using the procedure described in [27].

2. Image Streams Spatial and Temporal Registration

In this study, the reference 3D endoscopy images had different contrast from the 2D endoscopy scans. The MI was used to register the reference scan and endoscopy streams acquired at different frame rates, both spatially and temporally.

As illustrated in Figure S3, the foregrounds of two images acquired from different streams of the same sample were determined by brightness thresholding. Then the foregrounds were translated within a search range to discover corresponding landmarks from which the MI is maximized. After registering spatially each pair of images from the two streams, a mutual information matrix $\mathrm{MI} \in \mathrm{R}^{\mathrm{n_1}\times\mathrm{n_2}}$ was formed, wherein $\mathrm{n_1}$ and $\mathrm{n_2}$ were the number of image frames in each stream, respectively. The curve formed by the maxima of the MI in the matrix determined the temporal correspondence between the streams (Figure S4).

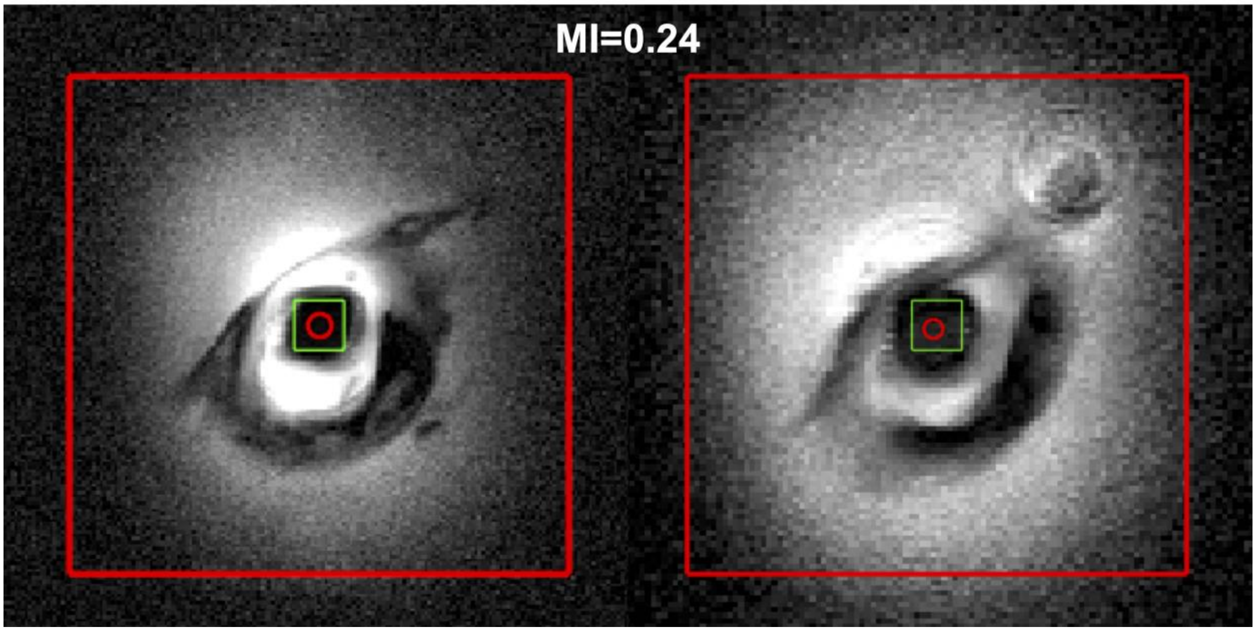

Figure S*3*. Left: image from a 3D static reference scan. Right: image from a 0.3 frame-per-second (fps) fully-sampled dynamic endoscopy scan. Red boxes show foreground patches. Green boxes are search region (5mm square) for translation. Red circles are corresponding landmarks found for registration.

3. Inter-frame Transitions in Real-time Endoscopy

To quantify the continuity of real-time image streams, the change of the location of the coil was treated as vectors over time. Considering scans from the same sample wherein the coil starts at location $\overrightarrow{L_0}$ and ends up at $\overrightarrow{L_T}$, we have:

$$\overrightarrow{L_T} = \int_0^T \overrightarrow{\Delta L_t} dt + \overrightarrow{L_0}$$

Assuming that real-time scans of the same sample follow similar paths, the average magnitude of $|\overrightarrow{\Delta L_t}|$ is inversely proportional to the smoothness of inter-frame transitions.

The average inter-frame translation distance (ΔL) of the center was calculated for 6fps and 10fps streams. The center of the foreground was determined by taking the weighted center of pixel values. The average translation distance was thus calculated as

$$\Delta L = \frac{1}{T-1} \sum_{t=2}^{T} \sqrt{(x_t - x_{t-1})^2 + (y_t - y_{t-1})^2} \tag{A2}$$

where x_t and y_t are coordinates of foreground center of the frame at dynamic t; and T is the number of frames in the image stream.

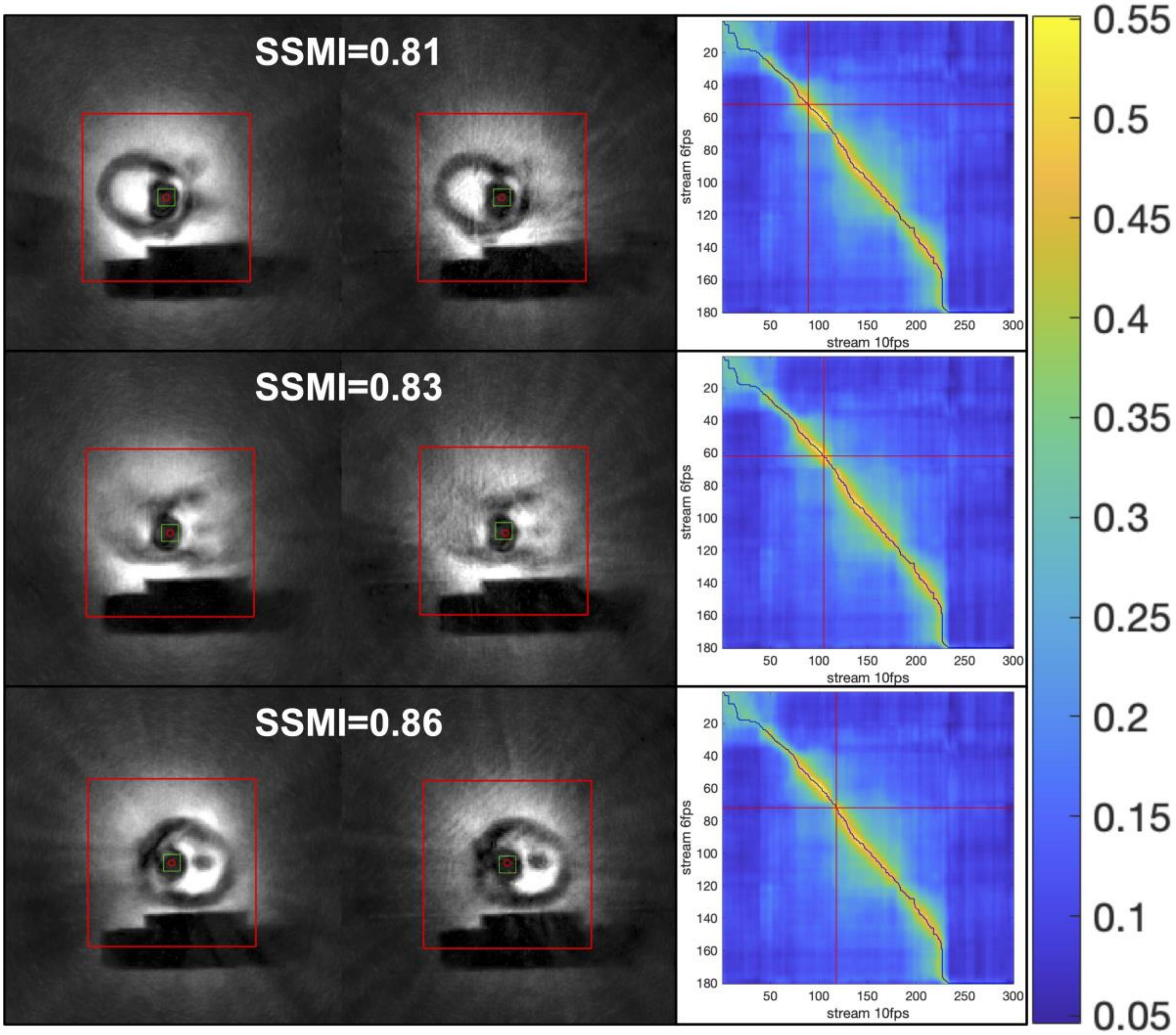

Figure S4. Example three dynamic scans from 6fps (left column) and 10fps (middle column) real-time endoscopy of an iliac artery when the coil transitions from a side branch (Fig. S1d) into the main lumen (top row: start transition; middle row: between the two lumens; bottom row: main lumen). Color maps (right column) show corresponding locations in the MI matrix of the temporally registered dynamics (vertical axis: dynamic indices of the 6fps stream; horizontal axis: dynamic indices of the 10fps stream). The blue curves are traces formed by MI maxima; red crosses show the specific image pairs

S4. MRI Endoscopy Videos

The corresponding videos of the MRI endoscopy images shown in Figure 2, 3, and 4 are provided online. Real-time endoscopy at 6fps, 10fps and conventional MRI endoscopy at 0.3fps are displayed at the true display speed as visualized on the scanner console.

Video S1 6fps real-time endoscopy of porcine vessel *ex vivo* in Figure 2 (row a).
Video S2 10fps real-time endoscopy of porcine vessel *ex vivo* in Figure 2 (row b).

Video S3 0.3fps fully-sampled “conventional” endoscopy of porcine vessel *ex vivo* in Figure 2 (row c).
Video S4 6fps real-time endoscopy of diseased human vessel *ex vivo* in Figure 3 (row a).
Video S5 10fps real-time endoscopy of diseased human vessel *ex vivo* in Figure 3 (row b).
Video S6 0.3fps fully-sampled “conventional” endoscopy of diseased human vessel *ex vivo* in Figure 3 (row c).
Video S7 6fps real-time endoscopy of porcine vessel *in vivo* in Figure 4 (row 1).
Video S8 10fps real-time endoscopy of porcine vessel *in vivo* in Figure 4 (row 2).
Video S9 0.3fps fully-sampled “conventional” endoscopy of porcine vessel *in vivo* in Figure 4 (row 3).
Video S10 Comparison of 6fps real-time endoscopy of diseased human vessel *ex vivo* in Figure 3 (row a) before and after preprocessing filter correction.
Video S11 Comparison of 10fps real-time endoscopy of porcine vessel *in vivo* in Figure 4 (row 2) before and after preprocessing filter correction.
